# Integrative illustration for coronavirus outreach

**DOI:** 10.1371/journal.pbio.3000815

**Published:** 2020-08-06

**Authors:** David S. Goodsell, Maria Voigt, Christine Zardecki, Stephen K. Burley

**Affiliations:** 1 RCSB Protein Data Bank, Rutgers, The State University of New Jersey, Piscataway, New Jersey, United States of America; 2 Department of Integrative Structural and Computational Biology, The Scripps Research Institute, La Jolla, California, United States of America; 3 Institute for Quantitative Biomedicine, Rutgers, The State University of New Jersey, Piscataway, New Jersey, United States of America; 4 Research Collaboratory for Structural Bioinformatics Protein Data Bank, San Diego Supercomputer Center, University of California, San Diego, California, United States of America; 5 Rutgers Cancer Institute of New Jersey, Rutgers, The State University of New Jersey, New Brunswick, New Jersey, United States of America

## Abstract

Two illustrations integrate current knowledge about severe acute respiratory syndrome (SARS) coronaviruses and their life cycle. They have been widely used in education and outreach through free distribution as part of a coronavirus-related resource at Protein Data Bank (PDB)-101, the education portal of the RCSB PDB. Scientific sources for creation of the illustrations and examples of dissemination and response are presented.

## Abstract 2

This Community Page article describes how visually striking illustrations of SARS coronaviruses can capture the state of knowledge of the field and provide a touchstone for outreach and education about these pathogens.

In this era of rapid and widespread flow of information, there is a persistent need for accurate and accessible scientific imagery. This is particularly true for the current pandemic of SARS-CoV-2 (severe acute respiratory syndrome coronavirus 2), where we have been bombarded with conflicting information that we must each reconcile into a coherent course of action to protect our own health. Early in the pandemic, we set the goal of creating integrative illustrations of the virus and its interactions with the cells that it infects for use at PDB-101, the education and outreach portal of the RCSB Protein Data Bank [1],[2], as part of a growing body of coronavirus resources [3]. The idea is to create coherent and self-consistent materials that synthesize the state of knowledge at a given time. Our hope is that these illustrations will help to put a face on the virus, to help us see that it is something that may be understood and fought, and that they will help present the viral and cellular context of topics being discussed in professional and popular media.

Images of viruses are ubiquitous in the scientific and popular media. For example, an internet image search of “HIV-1” will return all manner of illustrations, ranging from schematic cross-sections that show a parts list of components to highly rendered but often science-poor conceptual images for editorial venues. The US Centers for Disease Control and Prevention (CDC) released an exemplary computer graphics image of the SARS-CoV-2 virion early in the pandemic [4], with the forbidding white-and-red coloring scheme used in illustrations of HIV [5] and Ebola [6] from Visual Science. The CDC illustration, given its excellent provenance and close ties to primary data, has been widely used in the media and adapted in many derivative works.

In the virus imagery produced for PDB-101 [7], traditional methods of scientific illustration are used to create explanatory figures that reveal the inner structure of virions and explore their interactions with cells. These include educational features on HIV, Zika, Ebola, and others at PDB-101, as well as work on HIV and its life cycle with the HIV Interactions in Viral Evolution (HIVE) Center [8] and influenza with 3D Molecular Systems [9]. In this report, we present the scientific sources, integrative process, and public response for two illustrations of SARS coronaviruses distributed from PDB-101. As with other mesoscale landscape work, the illustrations depict cross-sections through the virus and cell, drawn at a consistent scale in orthographic projection, showing all macromolecules [10,11], and simplified shapes are used to represent biomolecular components to aid in recognition of molecules and comprehension of the scene as a whole. The illustrations integrate data that were available early in the coronavirus disease 2019 (COVID-19) pandemic, including atomic structures, if available, or molecular weights [12], and the ultrastructure is based on results from electron microscopy [13]. This information is provided in detail in S1 Text to assist with science communicators, educators, and students in their own work on SARS virus education and outreach.

*Coronavirus in Respiratory Mucosa* (Fig 1) shows a mature coronavirus virion in a respiratory context to provide a visual touchstone for understanding the virus as an entity that may be understood and fought [14]. It depicts a cross-section through coronavirus just entering the lungs, surrounded by mucus secreted by respiratory cells, secreted antibodies, and several small proteins from the innate immune system. The illustration was produced and released at the RCSB PDB on Feb 6, 2020, early in the epidemic and when the scientific community, the news media, and the general public were looking for information to understand the virus and its implications for world health. Publication coincided with the release of the first SARS-CoV-2 structure in the PDB archive, the main protease (PDB 6lu7).

**Fig 1:**
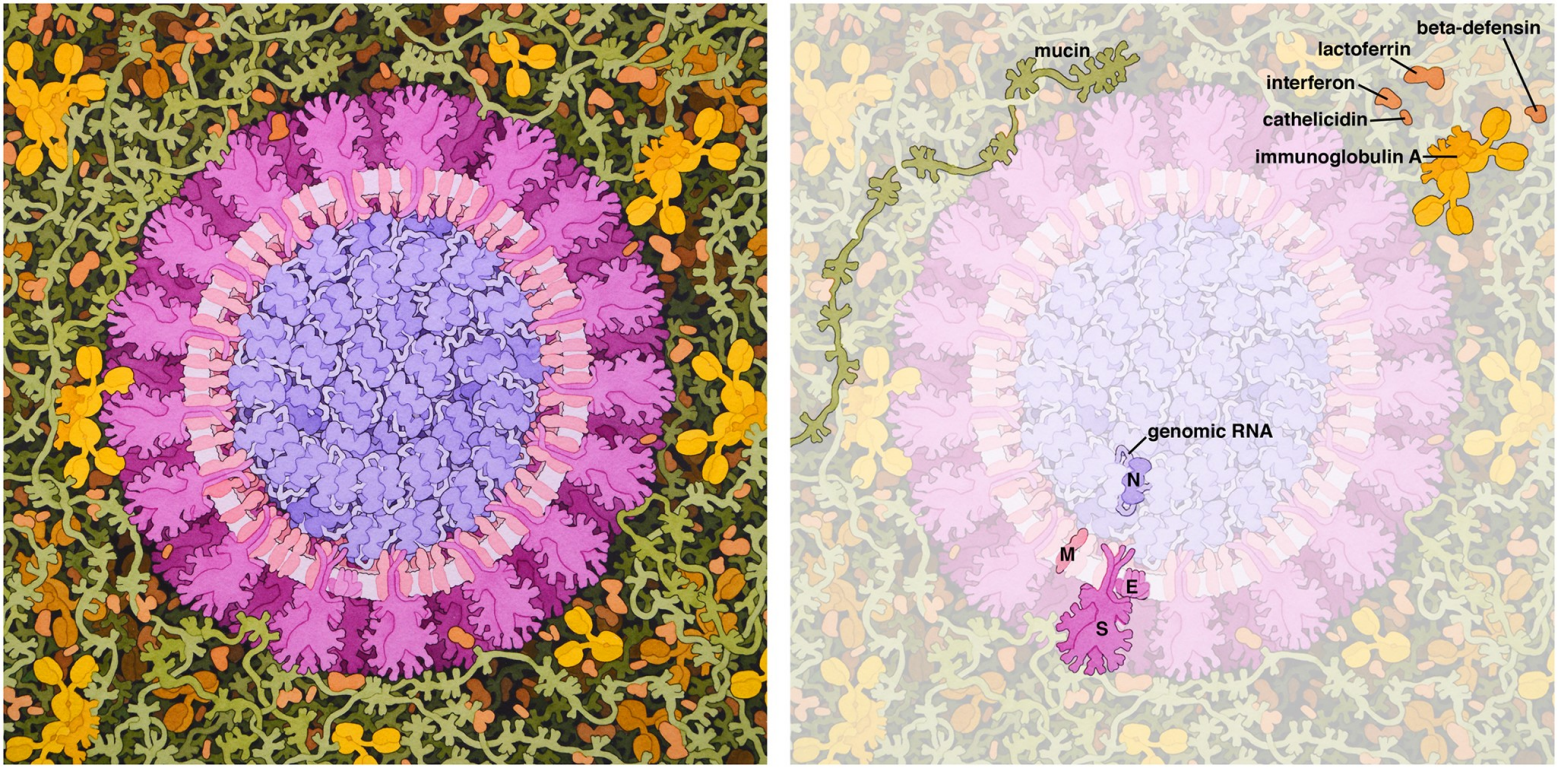
Coronavirus in respiratory mucosa. A cross section of the virus is shown, with membrane and membrane-bound viral proteins in magenta and the viral genome and associated proteins in purple. In the surrounding mucosa, mucins are shown in green and molecules of the immune system are in yellow and tan. Viral proteins are labeled: M, membrane protein; E, envelope protein; N, nucleocapsid protein; S, spike protein.

The goal of *Coronavirus Life Cycle* (Fig 2) is to show as many of the viral gene products as possible in action at a central moment in the viral life cycle to provide context as future drug design efforts begin reporting successes [15]. The illustration captures a time point when the virus is actively replicating in the cell. Reading from top to bottom, the overarching narrative includes synthesis of viral proteins at the endoplasmic reticulum (ER), replication of viral RNA by replicases on double membrane vesicles, and assembly and budding of virions into an endosome. In each of these three processes, many specific subprocesses are shown:

- at the top, viral RNA is directing the synthesis of viral spike proteins (Fig 3);
- in the middle, viral proteins have reorganized the ER membrane to form characteristic networks and double membrane vesicles;
- bound to these new membrane structures, several viral enzymes form replicase complexes that create new copies of the viral RNA genome (Fig 3);
- at the bottom, the viral nucleocapsid protein is condensing the RNA genome and interacting with the viral membrane protein to package the genome as the virus buds into an endosome.

**Fig 2:**
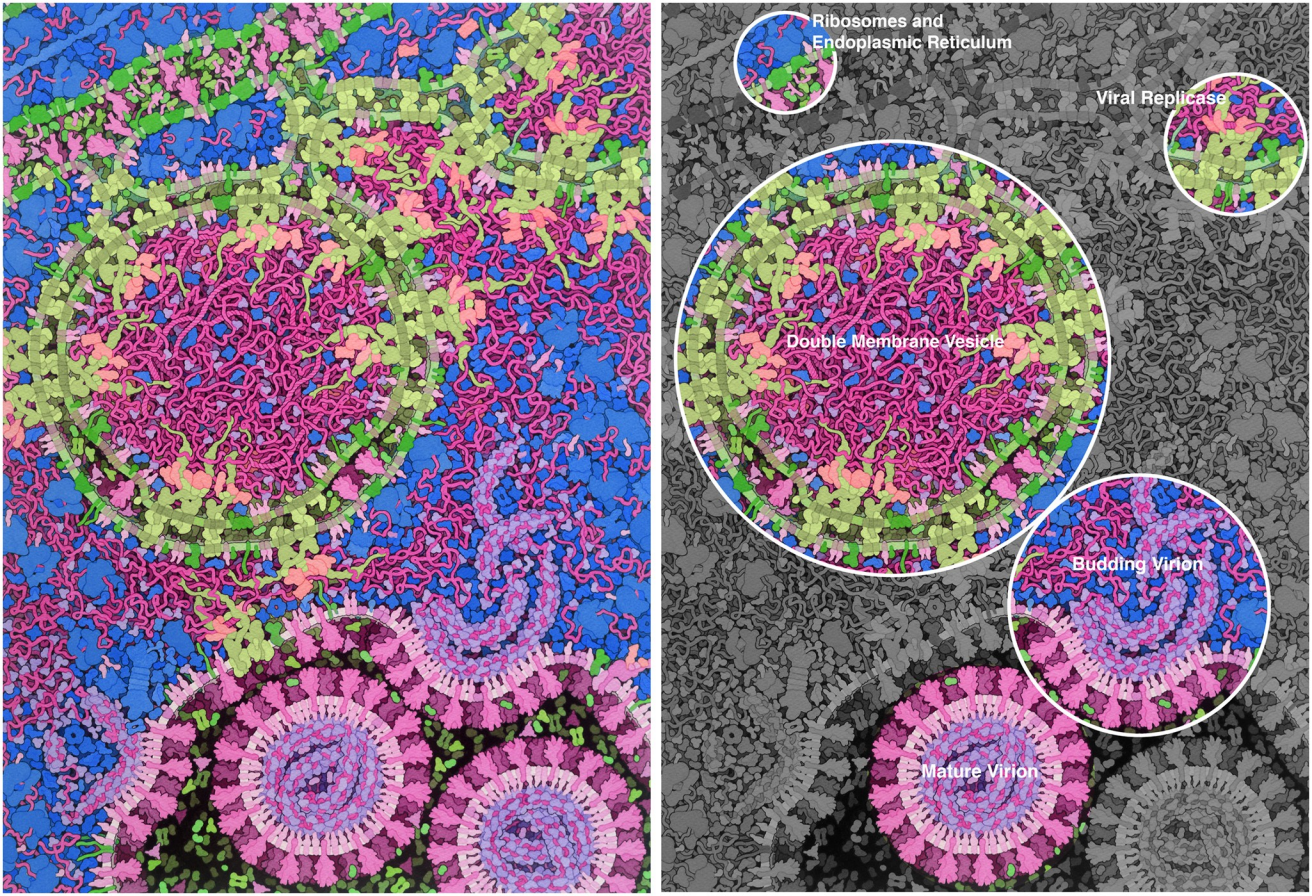
Coronavirus replication and budding. This illustration shows a cross-section through a cell infected with a coronavirus such as SARS-CoV-2. It shows a time point when the virus is actively replicating, and new viruses are being created. The cell’s molecules are shown in blues and greens, and the viral molecules are shown in reds and purples. The illustration integrates the current state of knowledge, but many aspects of the virus and its life cycle are still actively being studied, so portions of the illustration are speculative. Note that some features, such as RNA, needed to be slightly exaggerated in size/width, given the minimal size of features that could be depicted using black outlines of discernable width at the consistent magnification of 1,000,000× that was used for the original watercolor painting. SARS-CoV-2, severe acute respiratory syndrome coronavirus 2.

**Fig 3:**
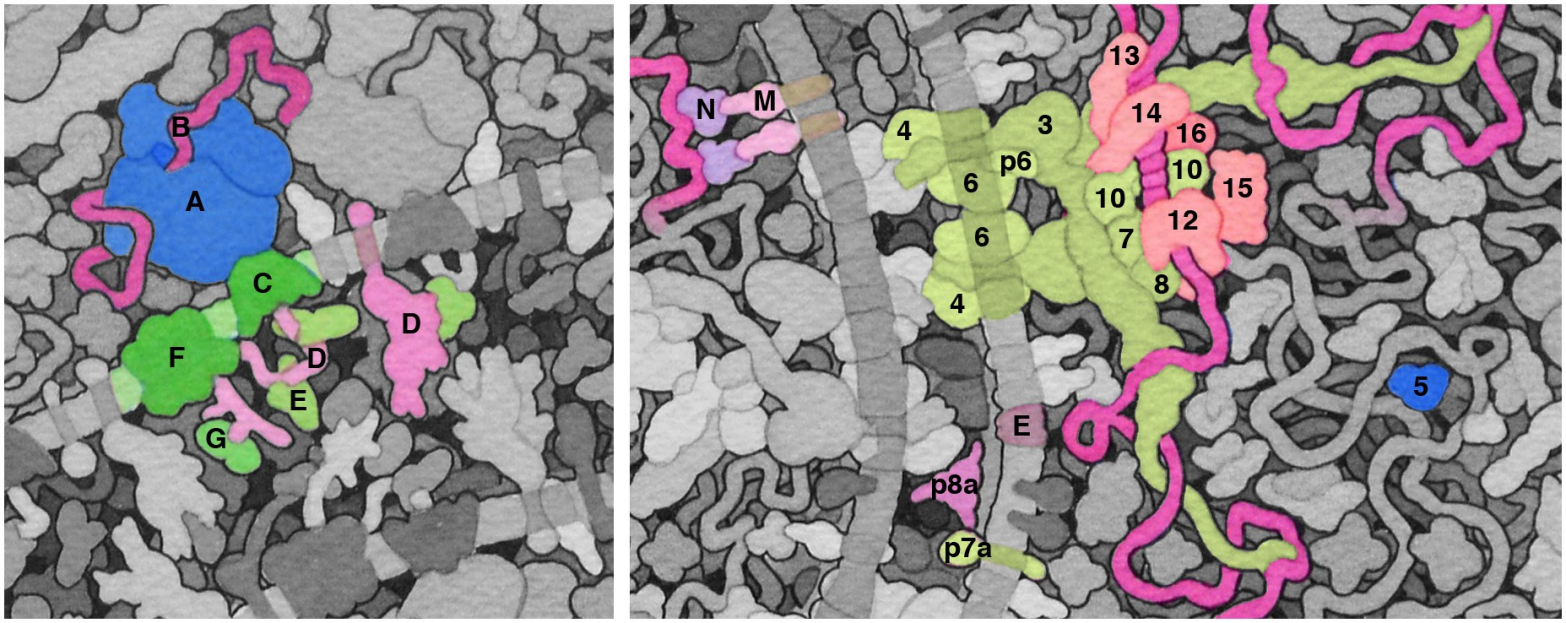
Details from coronavirus replication and budding. (Left) ribosomes in the endoplasmic reticulum. (A) ribosome; (B) viral coding RNA; (C) translocation channel; (D) spike protein; (E) chaperonin BiP; (F) oligosaccharide transferase; (G) glucosidases. (Right) viral proteins. Numbers identify the viral nsp, so “3” is nsp3. Three structural proteins (E, envelope; M, membrane; N, nucleocapsid), and several accessory proteins (p6, p7a, and p8a) are also shown. The sizes and shapes of proteins are based on structural results and known interactions, but the arrangement of subunits in the replicase is speculative. BiP, binding immunoglobulin protein; nsp, non-structural proteins.

The illustration is designed around one of the big mysteries of the coronavirus life cycle. The ER is extensively remodeled to form vesicles with two concentric membranes [16]. The outer membrane is thought to be connected to the ER, but the inner membrane is thought to be sealed shut. The vesicle is thought to include viral RNA, some of which may be in double-stranded form. The function of these vesicles is still a subject of study, with many questions to be answered. Is the RNA replicating inside? If so, how do the building blocks get into the vesicle, and how does the final product get out?

Both illustrations are available for free use under a Creative Commons CC-BY-4.0 license at PDB-101 [17] and are reaching a growing audience. As of the end of June 2020, the original coronavirus virion image had received >35,000 views. A black and white outline version of this image was distributed online for “coloring” and has been accessed >20,000 times. Other coronavirus-related materials offered at PDB-101 include a video demonstrating fighting coronavirus with soap at the molecular level (>360,000 views on YouTube) and a *Molecule of the Month* feature on *Coronavirus Proteases* (>68,000 views, [18]). The illustrations are also being used in research publications, for example, on the cover of the inaugural issue of *The Lancet Microbe* [19].

In addition to web usage statistics, social media and Twitter, in particular, provide a particularly useful glimpse into how the *Coronavirus in Respiratory Mucosa* illustration has been used by the general public, providing an anecdotal evaluation of its success. Unsolicited comments typically fall into several general categories. There was a disconnect for many viewers between the colorful approach of the illustration and the dangerous nature of the virus. Comments include:

- “This is beautiful and terrifying at the same time,”
- “Art is the work of transforming fear and pain into beauty,”

but also

- “Things seem a bit less scary when they’re colourful,”
- “So pretty that makes me forget how many lives it took…in one month!”

Art critic Phillip Kennicott [20] explored this concept more deeply, positing that the menacing colors of the CDC illustration “clearly emphasizes the threat this virus poses to those who refuse to, or cannot, socially distance themselves,” whereas the colorful watercolor depicts the virus as “a thing apart, to be studied, anatomized and understood.” Design of the color scheme was a particularly tricky aspect of the illustration. One of the design goals was to allow users to discern host molecules from components encoded by the virus. A scheme was chosen that is consistent with past work, with host features in blues and greens and viral features in purples and pinks. This, unfortunately, causes visual dissonance in, for example, the double-membrane vesicles that include both host and viral proteins. Perhaps this can be treated as a feature, however, not a problem, because the depicted scene is not a harmonious process but rather the forced hijacking of the host cell.

We have also received numerous comments from people using the illustration, and the accompanying coloring-book version of the illustration (Fig 4), in home schooling during the shelter-at-home period of the pandemic. A few of the (truly inspiring) comments include:

- “My younger daughter was just asking me last night, ‘do they know what the virus looks like?’”
- “Kids and I were talking today about how it is NOT an invisible enemy, just requires the right tools to see.”
- “My 6 yo was making himself sick with worry, not understanding why he soon won’t be able to go school. So I told him all about viruses, what they are and what they do, and sent him to school today with copies of this for his whole class.”

Many adults also made use of the coloring book version, as a “Relaxing and meditative evening activity—an appropriate ‘mandala’ for these days.”

**Fig 4:**
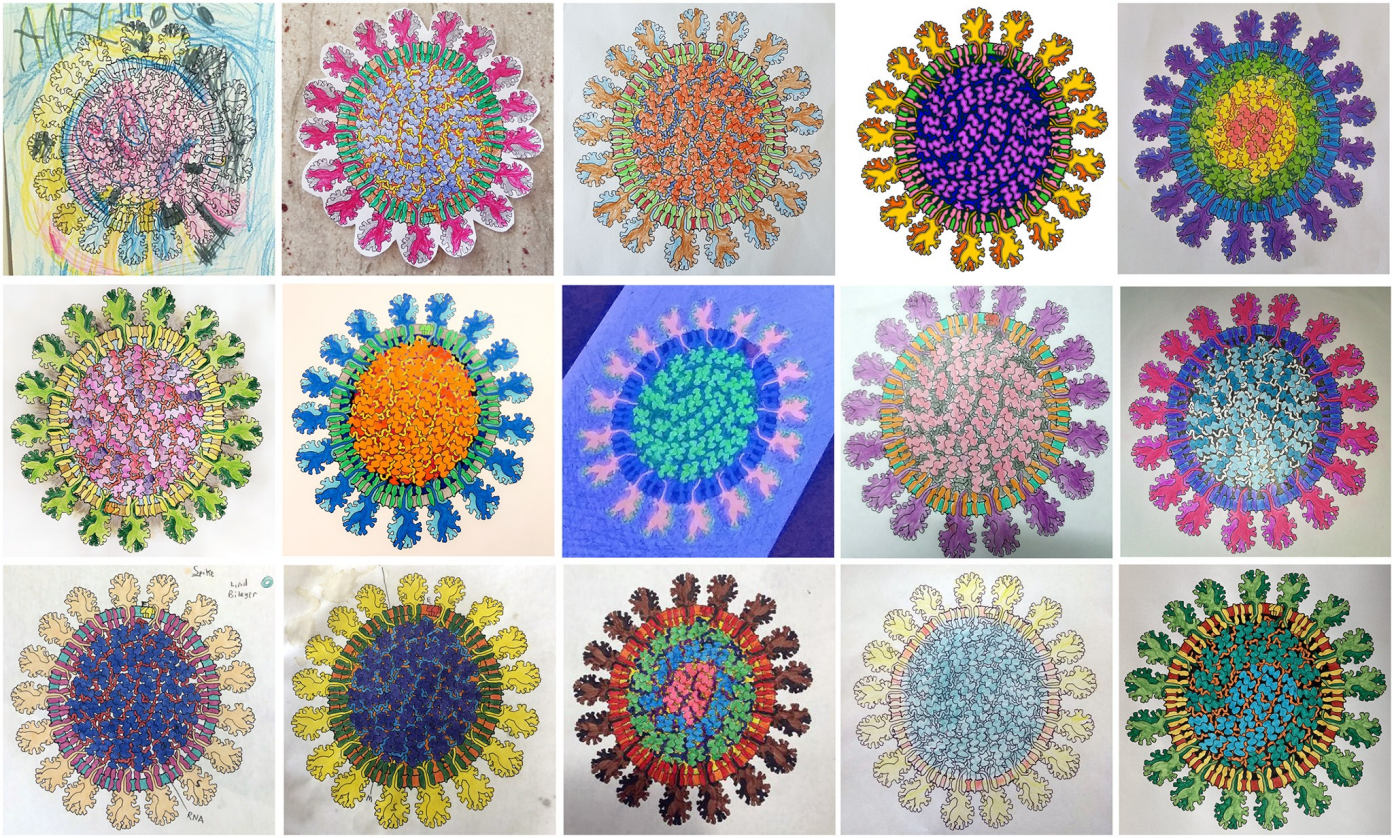
Selected online posts from the coronavirus coloring activity.

We have garnered several insights from this study, building on the direct contact that has been available through social media and interaction with feature writers and editors. The first is really no surprise but always good to revisit: different people respond differently to particular stylistic choices, in this case, to our coloring schemes. The intuitive color approach used here was received well by some and caused disconnects in others, and a lively online dialog with users led to recoloring of the replication illustration. These types of choices could use more careful study and tuning in future, for example, to address the needs of colorblind viewers; the challenge and fun is always finding the best mix of utility and artistic integrity. Our second insight is that fairly simple repurposing can extend the utility and reach of these types of integrative visualization efforts. For example, the coloring activity inspired readers to share related creations, including science-driven computer visualizations and 3D models as well as artistic interpretations in a variety of media.

As we write this article, sheltering at home, the research community is early in the fight against the new SARS-CoV-2, and a flood of new information is appearing daily. The structural biology community has been particularly engaged; since the release of the first main protease in February, nearly 150 structures from SARS-CoV-2 have been made available in the PDB archive. These illustrations represent snapshots of the information that was initially available and represent a generic depiction of the many coronavirus-related atomic structures. As our understanding of SARS-CoV-2 deepens, detailed modeling and depictions of this specific virus will become more feasible to help us chart a path through understanding the virus and its impact on global health.

## Supporting information

S1 Text Scientific sources. (DOCX)

## Abbreviations

CDC: Centers for Disease Control and Prevention
COVID-19: coronavirus disease 2019
ER: endoplasmic reticulum
HIVE: HIV Interactions in Viral Evolution Center
PDB: Protein Data Bank
RCSB: Research Collaboratory for Structural Bioinformatics
SARS: severe acute respiratory syndrome
SARS-CoV-2: severe acute respiratory syndrome coronavirus 2
