# Supplementary material for: Integrative illustration for coronavirus outreach
Source: PLoS Biol. 2020 Aug 6;18(8):e3000815. doi: 10.1371/journal.pbio.3000815 (PMC7433897; doi:10.1371/journal.pbio.3000815)
Supplement: S1 Text — (DOCX) [file pbio.3000815.s001.docx]

**Supporting Information: Scientific Sources**

These illustrations were produced early in the SARS-CoV-2 pandemic, so the illustrations are based primarily on information from SARS-CoV. Information is taken from published reports in peer-reviewed journals; planning for the illustration did not include personal communications from experts in the field.

Host Cell and Respiratory Mucosa. The host cell is based on a previous panorama through a B-cell [[1]](https://www.zotero.org/google-docs/?DM6wvR). The scene includes a small segment of the ER, with ribosomes attached to translocons, oligosaccharide transferase, chaperonins, etc. A simplified cytoplasm includes soluble enzymes and biomolecules involved in protein synthesis and a proteasome. A single actin filament is included near the ER. The respiratory mucosa is based on previous work with the [influenza life cycle](https://www.3dmoleculardesigns.com/Education-Products/Flu-Fight-Panorama.htm) (<https://www.3dmoleculardesigns.com/Education-Products/Flu-Fight-Panorama.htm>). Mucin-5B is depicted based on UniProt entry Q9HC84. IgA is depicted based on Ref. [[2]](https://www.zotero.org/google-docs/?XEu9iF) and molecules of the innate immune system [[3]](https://www.zotero.org/google-docs/?AbSPKQ) include beta-defensin (PDB 1e4s), lactoferrin (PDB 1bka), interferons (PDB 1itf) and small antimicrobial peptides like cathelicidin. Note, throughout this publication, four-character codes like this refer to accession codes in the PDB archive, and full citations for the structure determinations are available at any of the partner sites of the [wwPDB](http://www.wwpdb.org/) (<http://www.wwpdb.org/>).

Membranes. Coronaviruses such as SARS-CoV extensively remodel the cellular endomembrane system. The illustration is based largely on the summary in Figure 9 of Ref. [[4]](https://www.zotero.org/google-docs/?Qr5VcA), along with other sources [[5–7]](https://www.zotero.org/google-docs/?tMQeuA). Soon after infection, characteristic double-membrane vesicles (DMV) appear. They have been shown to be continuous with the ER. The vesicles typically have a diameter of 200-300 nm, and the spacing of the membranes is about 20 nm [[8]](https://www.zotero.org/google-docs/?8o4cRp). In addition, convoluted reticular membrane structures are also formed, which are thought to be sites of replication. At later stages, larger membrane bounded compartments are formed and are the site of budding of virions. These data were incorporated into the illustration to tell a temporal story from top to bottom. A segment of ER is shown at the top, with connections to a DMV of 250 nm diameter and connected to a region with a network of membrane tubules. A larger endosome is shown at the bottom. No connections between the DMV and endosome were shown, reflecting the speculative nature of the relationship between these structures.

RNA. The illustration depicts a time point when viral RNA is being actively replicated and translated. The site of replication is still a matter of some conjecture. Metabolic labeling and EM autoradiography studies see replication on/inside DMV [[9]](https://www.zotero.org/google-docs/?4E01lf) and immunoelectron microscopy studies see replication on the membrane structures associated with DMV and localize double-stranded RNA inside the DMV [[4]](https://www.zotero.org/google-docs/?ZhoH4w). To incorporate these data, active replication is depicted on the outside of DMV and on the associated membrane tubules. The DMV includes several (+) and (-) full genomes, partially paired to form dsRNA. No attempt was made to distinguish full genomes or subgenomic RNA. Coronavirus genomes are among the largest RNA virus genomes at ~30,000 bp, and would be roughly 10 meters long at this magnification, so showing an entire RNA strand is not feasible in the space of the illustration.

Structural Proteins. Spike (S) protein forms a trimer with a large ectodomain, a transmembrane segment, and a small intravirion domain [[10]](https://www.zotero.org/google-docs/?ABNYJd). The ectodomain is extensively glycosylated (UniProt P59594). CryoEM structures are available for the ectodomain, showing that the small receptor-binding domain at the tip of the spike is mobile, adopting both a closed conformation and an open conformation that is required for receptor binding [[11]](https://www.zotero.org/google-docs/?IGJVae). Electron micrographs show a dense packing of spikes on the surface, leading to the characteristic “crown” shape of the virus with 50-100 spike trimers for an averaged-size virion [[12]](https://www.zotero.org/google-docs/?RHye2e). In the illustrations, the shape of the ectodomain is based on a fully-closed complex (PDB 6vxx), although current results hypothesize that a dynamic equilibrium between open and closed forms might be one cause of the virulence of these viruses [[13]](https://www.zotero.org/google-docs/?e0Px9t).

The membrane (M) protein is involved in packaging the viral nucleoprotein into virions. No structures were available for the intact protein, so the illustration was based on structure predictions based on the sequence (UniProt P59596), which predict several transmembrane segments and an intravirion domain. This is also observed in cryoEM reconstructions, where an elongated domain is seen immediately inside the viral membrane [[12]](https://www.zotero.org/google-docs/?JYdh3n). Current estimates place roughly 1100 M proteins in the virion, which may represent dimers of the protein [[14]](https://www.zotero.org/google-docs/?p9wf8S). In the illustrations, M proteins were densely packed between spike proteins, and were associated with N proteins in the nucleoprotein to nod to their specific interaction.

Envelope (E) is thought to be involved in the process of budding [[15]](https://www.zotero.org/google-docs/?BVSCkv). Structures show a pentameric complex that forms a viroporin through the membrane (PDB 5x29), but the specific role that it plays is still a matter of speculation. A small number were placed in the mature virion, and several copies at the site of budding of the virus into the endosome.

The genomic RNA is condensed and packaged by the nucleocapsid (N) protein. Structures are available for the N-terminal domain (PDB 2ofz) and the C-terminal domain (PDB 2cjr), which forms a dimer. The C-terminal domain also forms an octamer in this crystal form, which has been hypothesized to be a model for a nucleoprotein filament [[16]](https://www.zotero.org/google-docs/?sygabX). Disordered regions link the domains and are present at the N- and C-terminus, and all portions show some RNA-binding ability, but the N-terminal domain and central linker are thought to play the major role [[17]](https://www.zotero.org/google-docs/?AWaT7C). Gently-isolated nucleoproteins often show a rope-like form with multiple beads, typically about 15 nm wide [[18]](https://www.zotero.org/google-docs/?MlU8WM). Similar beads are seen to form a locally-ordered array under the layer of M protein in cryoEM reconstructions [[12]](https://www.zotero.org/google-docs/?z6GXCD). Current estimates place roughly 730-2200 nucleocapsid chains in the virion, which corresponds to about 60 RNA nucleotides per N dimer [[14]](https://www.zotero.org/google-docs/?MIx4jn). These data were interpreted in two slightly different ways in the illustrations. In both cases, the dimer was treated as the basic unit, and a simplified representation was used that has the two domains loosely associated with each other, not depicting the disordered regions explicitly. In the virion image, dimers were stacked in lines that followed the interior contour of the membrane. In the lifecycle image, dimers were associated into rope-like assemblies, which were then folded into the virion.

Replicase. The viral replicase is synthesized as two variations of a long polyprotein, which are then clipped into functional non-structural proteins (abbreviated “nsp”) by the two viral proteases. The illustration is based primarily on a comprehensive review [[19]](https://www.zotero.org/google-docs/?qHUFD0). Most of these proteins are thought to assemble into one or more complexes that replicate and proofread the viral RNA and create a cap at the 5’ end. For the illustration, information on the structure and interactions of each component were used to define sizes and shapes, but the form of the final assembly is speculative. Nsp1 suppresses host gene expression and the function of nsp2 is not known, so they were not included in the illustration. Nsp5 is the main protease that cuts the viral polyproteins into functional pieces. Extensive structural information is available for this protein, since it is a major target for discovery of new therapeutics. It forms a heart-shaped soluble dimer (PDB 1q2w).

Nsp3 is the largest non-structural protein, with multiple functional domains [[20]](https://www.zotero.org/google-docs/?MMbMDJ), including two transmembrane segments near the C-terminus. Structures are available for many of these domains. Several features were included in the illustration [[19]](https://www.zotero.org/google-docs/?mgyh8z): the N-terminal Ubl1 domain binds to ssRNA binding and is connected with flexible linker; the NAB (nucleic acid binding domain) forms a dimer; the NAB-betaSM-TM1 domains interact with nsp5, 7, 8, and 12-16; and the C-terminal CoV-Y domain interacts with nsp9 and 12. In the illustration, the membrane-bound dimer of nsp3 was depicted with multiple flexibly-connected domains and associated with other replicase components through the NAB and other C-terminal domains. Two other membrane-bound proteins, Nsp4 and 6, are membrane-spanning proteins that remodel the ER membrane and mediate the formation of DMV [[8,21]](https://www.zotero.org/google-docs/?hOl9bU). No structural information was available at the time, so the size of the structures and their many transmembrane segments were based on annotations in UniProt P0C6U8. Speculative interactions of these proteins were shown in the illustration bridging the two membranes in the DMV.

The replicase enzymes perform the synthetic tasks of replication and capping. Nsp12, the RNA-directed RNA polymerase, creates new viral RNA strands (PDB 6nur). Nsp13 is a helicase that separates strands in an RNA double helix (PDB 6jyt). Nsp14 is a guanine N7-methytransferase that includes an exoribonuclease involved in proofreading (PDB 5c8s). Nsp15 is a uridylate-specific endoribonuclease that breaks RNA, the function of which is still under study. It is seen as a stable hexamer in PDB entry 6w01. It is included as part of the replicase in the illustration and also near the top of the illustration degrading excess RNA. Nsp16 is a 2’-O-methyltransferase that is involved in formation of the RNA cap (PDB 6w61). Nsp7, 8, and 10 are proteins involved in organizing the replicase complex [[22]](https://www.zotero.org/google-docs/?uwc5ZR). Interaction of nsp7 and nsp8 with the polymerase nsp12 was based on PDB entry 6nur, and interaction of nsp10 with nsp16 and nsp14 was based on PDB entries 6w61 and 5c8s. In the illustration, a dimer of nsp10 was included [[23]](https://www.zotero.org/google-docs/?n4m0Y5).

Accessory proteins. Several accessory proteins (p6, p7a, p8ab, p9b) are also shown, based on a recent review [[24]](https://www.zotero.org/google-docs/?W1OGbj). These are dispensable for replication of the virus, but are involved in the virulence of infection. P6 is a small protein amphipathic protein that binds on the cytoplasmic side of ER/Golgi membranes, associated with nsp3 and nsp6. P7a is also bound to the ER with one transmembrane segment and a large domain on the lumenal side. P8ab is a small protein that forms trimers inside the ER, with one site of glycosylation. P9b is a small protein that interacts with several other components, including E. Other accessory proteins are thought to perform their jobs in other parts of the cell (Golgi, nucleolus, etc.) and are not included here.

**REFERENCES**

[1. Goodsell DS. Miniseries: Illustrating the machinery of life: Eukaryotic cell panorama. Biochem Mol Biol Educ. 2011;39: 91–101. doi:10.1002/bmb.20494](https://www.zotero.org/google-docs/?MNa2Qx)

[2. Bonner A, Almogren A, Furtado PB, Kerr MA, Perkins SJ. The nonplanar secretory IgA2 and near planar secretory IgA1 solution structures rationalize their different mucosal immune responses. J Biol Chem. 2009;284: 5077–5087. doi:10.1074/jbc.M807529200](https://www.zotero.org/google-docs/?MNa2Qx)

[3. Rogan MP, Geraghty P, Greene CM, O’Neill SJ, Taggart CC, McElvaney NG. Antimicrobial proteins and polypeptides in pulmonary innate defence. Respir Res. 2006;7: 29. doi:10.1186/1465-9921-7-29](https://www.zotero.org/google-docs/?MNa2Qx)

[4. Knoops K, Kikkert M, Worm SHE van den, Zevenhoven-Dobbe JC, van der Meer Y, Koster AJ, et al. SARS-coronavirus replication is supported by a reticulovesicular network of modified endoplasmic reticulum. PLoS Biol. 2008;6: e226. doi:10.1371/journal.pbio.0060226](https://www.zotero.org/google-docs/?MNa2Qx)

[5. Goldsmith CS, Tatti KM, Ksiazek TG, Rollin PE, Comer JA, Lee WW, et al. Ultrastructural characterization of SARS coronavirus. Emerg Infect Dis. 2004;10: 320–326. doi:10.3201/eid1002.030913](https://www.zotero.org/google-docs/?MNa2Qx)

[6. Neuman BW, Angelini MM, Buchmeier MJ. Does form meet function in the coronavirus replicative organelle? Trends Microbiol. 2014;22: 642–647. doi:10.1016/j.tim.2014.06.003](https://www.zotero.org/google-docs/?MNa2Qx)

[7. Harak C, Lohmann V. Ultrastructure of the replication sites of positive-strand RNA viruses. Virology. 2015;479–480: 418–433. doi:10.1016/j.virol.2015.02.029](https://www.zotero.org/google-docs/?MNa2Qx)

[8. Angelini MM, Akhlaghpour M, Neuman BW, Buchmeier MJ. Severe Acute Respiratory Syndrome Coronavirus Nonstructural Proteins 3, 4, and 6 Induce Double-Membrane Vesicles. Moscona A, editor. mBio. 2013;4: e00524-13. doi:10.1128/mBio.00524-13](https://www.zotero.org/google-docs/?MNa2Qx)

[9. Snijder EJ, Limpens RWAL, de Wilde AH, de Jong AWM, Zevenhoven-Dobbe JC, Maier HJ, et al. A unifying structural and functional model of the coronavirus replication organelle: tracking down RNA synthesis. Microbiology; 2020 Mar. doi:10.1101/2020.03.24.005298](https://www.zotero.org/google-docs/?MNa2Qx)

[10. Li F. Structure, Function, and Evolution of Coronavirus Spike Proteins. Annu Rev Virol. 2016;3: 237–261. doi:10.1146/annurev-virology-110615-042301](https://www.zotero.org/google-docs/?MNa2Qx)

[11. Kirchdoerfer RN, Wang N, Pallesen J, Wrapp D, Turner HL, Cottrell CA, et al. Stabilized coronavirus spikes are resistant to conformational changes induced by receptor recognition or proteolysis. Sci Rep. 2018;8: 15701. doi:10.1038/s41598-018-34171-7](https://www.zotero.org/google-docs/?MNa2Qx)

[12. Neuman BW, Adair BD, Yoshioka C, Quispe JD, Orca G, Kuhn P, et al. Supramolecular Architecture of Severe Acute Respiratory Syndrome Coronavirus Revealed by Electron Cryomicroscopy. J Virol. 2006;80: 7918–7928. doi:10.1128/JVI.00645-06](https://www.zotero.org/google-docs/?MNa2Qx)

[13. Walls AC, Park Y-J, Tortorici MA, Wall A, McGuire AT, Veesler D. Structure, Function, and Antigenicity of the SARS-CoV-2 Spike Glycoprotein. Cell. 2020;181: 281-292.e6. doi:10.1016/j.cell.2020.02.058](https://www.zotero.org/google-docs/?MNa2Qx)

[14. Neuman BW, Kiss G, Kunding AH, Bhella D, Baksh MF, Connelly S, et al. A structural analysis of M protein in coronavirus assembly and morphology. J Struct Biol. 2011;174: 11–22. doi:10.1016/j.jsb.2010.11.021](https://www.zotero.org/google-docs/?MNa2Qx)

[15. Schoeman D, Fielding BC. Coronavirus envelope protein: current knowledge. Virol J. 2019;16: 69. doi:10.1186/s12985-019-1182-0](https://www.zotero.org/google-docs/?MNa2Qx)

[16. Chang C, Chen C-MM, Chiang M, Hsu Y, Huang T. Transient Oligomerization of the SARS-CoV N Protein – Implication for Virus Ribonucleoprotein Packaging. Menéndez-Arias L, editor. PLoS ONE. 2013;8: e65045. doi:10.1371/journal.pone.0065045](https://www.zotero.org/google-docs/?MNa2Qx)

[17. Chang C, Hou M-H, Chang C-F, Hsiao C-D, Huang T. The SARS coronavirus nucleocapsid protein – Forms and functions. Antiviral Res. 2014;103: 39–50. doi:10.1016/j.antiviral.2013.12.009](https://www.zotero.org/google-docs/?MNa2Qx)

[18. Macneughton MR, Davies HA. Ribonucleoprotein-like structures from coronavirus particles. J Gen Virol. 1978;39: 545–549. doi:10.1099/0022-1317-39-3-545](https://www.zotero.org/google-docs/?MNa2Qx)

[19. Neuman BW, Chamberlain P, Bowden F, Joseph J. Atlas of coronavirus replicase structure. Virus Res. 2014;194: 49–66. doi:10.1016/j.virusres.2013.12.004](https://www.zotero.org/google-docs/?MNa2Qx)

[20. Lei J, Kusov Y, Hilgenfeld R. Nsp3 of coronaviruses: Structures and functions of a large multi-domain protein. Antiviral Res. 2018;149: 58–74. doi:10.1016/j.antiviral.2017.11.001](https://www.zotero.org/google-docs/?MNa2Qx)

[21. Neuman BW. Bioinformatics and functional analyses of coronavirus nonstructural proteins involved in the formation of replicative organelles. Antiviral Res. 2016;135: 97–107. doi:10.1016/j.antiviral.2016.10.005](https://www.zotero.org/google-docs/?MNa2Qx)

[22. Snijder EJ, Decroly E, Ziebuhr J. The Nonstructural Proteins Directing Coronavirus RNA Synthesis and Processing. Advances in Virus Research. Elsevier; 2016. pp. 59–126. doi:10.1016/bs.aivir.2016.08.008](https://www.zotero.org/google-docs/?MNa2Qx)

[23. Subissi L, Imbert I, Ferron F, Collet A, Coutard B, Decroly E, et al. SARS-CoV ORF1b-encoded nonstructural proteins 12–16: Replicative enzymes as antiviral targets. Antiviral Res. 2014;101: 122–130. doi:10.1016/j.antiviral.2013.11.006](https://www.zotero.org/google-docs/?MNa2Qx)

[24. Liu DX, Fung TS, Chong KK-L, Shukla A, Hilgenfeld R. Accessory proteins of SARS-CoV and other coronaviruses. Antiviral Res. 2014;109: 97–109. doi:10.1016/j.antiviral.2014.06.013](https://www.zotero.org/google-docs/?MNa2Qx)
